# Type 1 and type 2 diabetes in celiac disease: prevalence and effect on clinical and histological presentation

**DOI:** 10.1186/s12876-016-0488-2

**Published:** 2016-07-25

**Authors:** Antti Kylökäs, Katri Kaukinen, Heini Huhtala, Pekka Collin, Markku Mäki, Kalle Kurppa

**Affiliations:** 1 School of Medicine, University of Tampere, Tampere, Finland; 2 Department of Internal Medicine, Tampere University Hospital, Tampere, Finland; 3 Tampere School of Health Sciences, University of Tampere, Tampere, Finland; 4 Department of Gastroenterology and Alimentary Tract Surgery, Tampere University Hospital, Tampere, Finland; 5 Tampere Centre for Child Health Research, University of Tampere and Tampere University Hospital, Biokatu 10, 33520 Tampere, Finland

**Keywords:** Celiac disease, Type 1 diabetes, Type 2 diabetes, Co-morbidity

## Abstract

**Background:**

Association between celiac disease and type 1 diabetes in adults is still somewhat unclear, and that between celiac disease and type 2 diabetes even less known. We studied these issues in a large cohort of adult celiac disease patients.

**Methods:**

The prevalence of type 1 and type 2 diabetes in 1358 celiac patients was compared with the population-based values. Furthermore, patients with celiac disease and concomitant type 1 or type 2 diabetes and those with celiac disease only underwent comparisons of clinical and histological features and adherence to gluten-free diet.

**Results:**

The prevalence of type 1 diabetes (men/women) was 8.0 % /1.8 % in celiac patients and 0.7 % /0.3 % in the population, and that of type 2 diabetes 4.3 % /2.5 % and 4.4 % /3.0 %, respectively. Celiac patients with concomitant type 1 diabetes were younger (45 years *vs* 65 years and 52 years, *P* < 0.001) and more often screen-detected (43 % *vs* 13 % and 14 %, *P* < 0.001), had less other gastrointestinal diseases (8 % *vs* 40 % and 25 %, *P* = 0.028), more thyroidal diseases (18 % *vs* 16 % and 13 %, *P* = 0.043) and lower dietary adherence (71 % *vs* 95 % and 96 %, *P* < 0.001) compared with celiac patients with concomitant type 2 diabetes and patients with celiac disease only. Patients with concomitant type 2 diabetes had more hypercholesterolemia than the other groups (8 % *vs* 6 % and 4 %, *P* = 0.024), and both diabetes groups more hypertension (47 % and 31 % vs 15 %, *P* < 0.001) and coronary artery disease (29 % and 18 % *vs* 3 %, *P* < 0.001) than the patients with celiac disease only.

**Conclusions:**

Type 1 diabetes was markedly overrepresented in celiac disease, especially in men, whereas the prevalence of type 2 diabetes was comparable with the population. Concomitant type 1 or type 2 diabetes predisposes celiac patients to severe co-morbidities and type 1 diabetes also to poor dietary adherence.

## Background

Celiac disease is a life-long immune-mediated disorder characterized by gluten-triggered inflammation and morphological damage of the small-bowel mucosa in genetically susceptible individuals [1, 2]. The disease is one of the commonest food-related disorders with a currently estimated prevalence of 1–2 % in the Western populations [3]. Moreover, it is overrepresented in type 1 diabetes (DM1), which shares a mutual genetic predisposition with CD [4]. Although this association is well established particularly in children, the effect of concomitant DM1 on the clinical and histological presentation in adult celiac disease patients remains unclear. In contrast to DM1, the non-autoimmune-mediated type 2 diabetes (DM2) is not considered to be overrepresented in celiac disease [5]. In fact, Kabbani and colleagues [6] have recently reported DM2 to be even less frequent among celiac disease patients than in the US population in general, indicating possible protective effect. Evidently, their finding should be confirmed in other countries. In addition, the effect of concomitant DM2 on the presentation and natural history of celiac disease in adult patients is scarcely studied.

The aim of the present study was to investigate the prevalence of DM1 and DM2 in Finnish adults with celiac disease and to compare these figures with those in the general population. In addition, we compared a variety of clinical, serological and histological parameters and dietary adherence between celiac disease patients with and without concomitant DM1 or DM2.

## Methods

### Patients and study design

The study was conducted at the University of Tampere and Tampere University Hospital. The celiac disease cohort comprised of 1358 adults (984 women and 374 men, age at diagnosis ≥16 years) collected from our regularly updated research database. The patient information was gathered from the medical records and, when inadequate, supplemented with personal interviews by a physician or study nurse. Following information at celiac disease diagnosis was collected from each subject: demographic, clinical and histological data, blood hemoglobin and celiac disease serology, presence of concomitant other chronic medical conditions, family history of celiac disease, and adherence to the gluten-free diet. The diagnosis of celiac disease was based on the demonstration of small-bowel mucosal villous atrophy and crypt hyperplasia in duodenal biopsies taken upon gastroscopy [2]. After the analyses, the patients were divided into three groups based on the presence of celiac disease and either concomitant DM1 (DM1 group), DM2 (DM2 group), or no diabetes (CD group). The diagnoses of DM1 and DM2 were set by the hospital physicians according to the national guidelines [7].

The prevalence of DM1 and DM2 was compared with that of general population utilizing the results of the Health 2000 survey [8]. The survey consists of 7419 Finnish adults aged ≥30 years who participated on a nationwide public health study. From these 6354 (79.7 %) underwent further examination at primary care where the presence of self-reported diabetes was confirmed. Next, these nationwide representative prevalences were compared with those found in the present study. Due to the age limitation of the Health 2000 study [9] only celiac disease patients aged ≥30 years were included in this comparison (*n* = 1254).

The Regional Ethics Committee of the Tampere University Hospital District approved the study protocol, and all subjects participating to the personal interviews gave written informed consent.

### Clinical presentation, serology and adherence to gluten-free diet

The main mode of presentation at celiac disease diagnosis was categorized into three groups: 1. Classical gastrointestinal celiac disease comprising subjects with for example diarrhea and malabsorption; 2. non-classical presentations such as dermatitis herpetiformis, infertility, joint pains and neurological symptoms; and 3. apparently silent/asymptomatic subjects detected by screening in at-risk groups. From the concomitant chronic diseases, particular attention was paid to the presence of autoimmune and gastrointestinal disorders, osteoporosis or osteopenia, asthma, malignancies, hypercholesterolemia and cardiovascular diseases. Anemia at diagnosis was defined as a hemoglobin value <13.4 g/dL in men and <11.7 g/dL in women [9].

Adherence to the gluten-free diet was considered “strict” if the patient reported no dietary lapses. A few minor lapses less than once per a month was regarded as “occasional lapses”, and lapses more often than that as “no gluten-free diet”. Furthermore, the strictness of the diet was estimated on the basis of the positivity (titer 1:≥5) to endomysial antibodies (EmA) after a minimum of one year on gluten-free diet. The antibodies were measured by a well-validated in-house test that uses human umbilical cord as substrate [10].

### Small-bowel mucosal histology

In our settings 4–6 duodenal biopsies are routinely taken in each case with celiac disease suspicion. The specimens are further referred to the hospital pathology department where the degree of small-bowel mucosal damage is evaluated from well-orientated biopsy cuttings [11]. Here the severity of the mucosal lesion was graded to partial, subtotal or total villous atrophy based on the original pathology report. Further, the presence of possible repeat biopsy and the degree of mucosal recovery on a gluten-free diet were recorded.

### Availability of data and materials

All available raw data will not be shared as this contains confidential patient information. All other relevant study data are presented in the Tables.

### Statistics

Variables are presented either as percentages or medians with range. Categorical variables were analyzed using chi-square and Fisher’s exact test, and numeric variables using the Kruskal –Wallis test. Logistic regression was used to adjust for gender, current age and age at the celiac disease diagnosis when appropriate. These results are presented in the Table 2 as “Crude” referring to the results of chi-square or Fisher’s exact test and as “Adjusted” referring to those of logistic regression. P value ≤ 0.05 was considered significant. All analyses were executed using Statistical Package for the Social Sciences version 20 (IBM, Armonk, NY, USA).

## Results

### Prevalence of concomitant type 1 and type 2 diabetes in celiac disease

Of the all 1358 celiac disease patients, 51 (3.8 %) had a concomitant DM1 and 38 (2.8 %) DM2. The corresponding figures in those aged ≥30 years (*n* = 1254) were 44 (3.5 %) and 38 (3.0 %), respectively. In this age group, the prevalence of DM1 was markedly higher in celiac patients than in the general population (Table 1). In men, DM1 was particularly overrepresented in celiac patients aged 30–64 years, and somewhat less in those aged over 65 years. In women DM1 was roughly five times more common in celiac disease than in the population in patients aged ≥30 years. In contrast to DM1, DM2 was slightly underrepresented in celiac men ≥65 years, and there was no marked difference between celiac disease and population in younger men or women (Table 1).

**Table 1:** Prevalence of type 1 and type 2 diabetes in celiac disease^a^ and general population^b^

			DM1		DM2	
			Celiac disease	General population	Celiac disease	General population
	Age	Sex	%	%	%	%
All	≥30	M	8.0	0.7	4.3	4.4
		F	1.8	0.3	2.5	3.0
Subgroups	30–64	M	10.0	0.7	3.8	3.0
		F	1.9	0.4	1.2	1.5
	≥ 65	M	2.2	0.8	5.6	9.4
		F	1.1	0.2	7.9	8.9

DM1, type 1 diabetes; DM 2, type 2 diabetes

^a^Study patients with celiac disease aged 30 years or more (*n* = 1254)

^b^The Health 2000 Survey

### Demographic data and clinical features at celiac disease diagnosis

The DM1 group comprised lower proportion of women than the DM2 group and CD groups. The median age at celiac disease diagnosis was higher in DM2 group than in the two other groups (Table 2). DM1 patients had less abdominal symptoms and malabsorption, and they were more often detected by serologic screening than the subjects in the DM2 and CD groups. There were no significant differences between the groups in the severity of histological damage and hemoglobin levels or presence of anemia, percentages of celiac disease in relatives and positivity to EmA (Table 2).

**Table 2:** Clinical, serological and histological characteristics of celiac disease in 1358 patients^a^

	DM1	DM2	No diabetes	
	*n* = 51	*n* = 38	*n* = 1269	P value
Females, %	39	61	74	<0.001
Current age, median (range), yr	45 (18–71)	65 (45–83)	52 (20–92)	<0.001
Age at dg of CD, median (range), yr	38 (2–70)	53 (26–76)	42 (2–89)	<0.001
Mode of presentation at dg, %				<0.001
Gastrointestinal symptoms	35	50	54	
Malabsorption	8	24	17	
Extra-intestinal symptoms^b^	14	13	16	
Risk-group screening^c^	43	13	14	
Hb at dg, median (range), g/l	131 (114–152)	134 (112–165)	128 (39–174)	0.272
Anemia at dg, %	20	26	27	0.515
CD in relatives, %	58	61	60	0.964
Positive EmA at dg, %	100	91	88	0.431
Degree of villous atrophy, %				0.433
Total	42	36	31	
Subtotal	29	50	38	
Partial	29	14	31	

DM1, type 1 diabetes; DM2, type 2 diabetes

*Hb* hemoglobin, *EmA* endomysial antibodies

^a^Celiac disease patients with or without concomitant DM1 or DM2

^b^Dermatitis herpetiformis, infertility, arthralgia or arthritis, osteoporosis, dental enamel defects, polyneuropathy, hypertransaminasemia, aphtous stomatitis

^c^DM1, family history for celiac disease, autoimmune thyroid disease, Sjögren’s syndrome

Concomitant DM1 or DM2 increased the risk of coronary artery disease and hypertension (Table 3). Furthermore, patients in the DM1 group had more thyroidal diseases and less other gastrointestinal diseases except celiac disease, and those in the DM2 group more hypercholesterolemia compared with the two other groups. There were no significant differences in the prevalence of osteoporosis, asthma or malignancies between the groups (Table 3).

**Table 3:** Presence of co-morbidities and adherence to the gluten-free diet (GFD) in 1358 celiac disease patients^a^

	DM1	DM2	No diabetes	P value	
	*n* = 51	*n* = 38	*n* = 1269	Crude^b^	Adjusted^c^
Co-morbidity, %					
Autoimmune thyroidal diseases	18	16	13	0.538	0.043
Coronary artery disease	18	29	3	<0.001	<0.001
Hypertension	31	47	15	<0.001	<0.001
Hypercholesterolemia	8	24	6	0.001	0.024
Osteoporosis or osteopenia	4	11	13	0.165	0.225
Asthma	4	13	7	0.265	0.456
Malignancy^d^	4	5	4	0.815	0.873
Gastrointestinal disease^e^	8	40	25	0.002	0.028
GFD, median (range), yr	6 (1–36)	7 (1–32)	8 (1–59)	0.935	
Strictness of GFD, %				<0.001	
Strict	71	95	96		
Occasional gluten	22	5	4		
No diet	6	0	0		
Positive EmA on GFD, %	30	9	6	<0.001	<0.001

DM1, type 1 diabetes; DM2, type 2 diabetes

*EmA* endomysial antibodies

^a^Celiac disease patients with or without concomitant DM1 or DM2

^b^Chi-square and Fisher’s exact test

^c^Adjusted for current age, age at celiac disease diagnosis and gender by logistic regression

^d^Cancer of breast, prostate, colon, small intestine, uterus, thyroid gland, ovary, lip, spine; sarcoma

^e^Lactose intolerance, food allergy, gastroesophageal reflux disease, Barrett’s esophagus, gastric ulcer, diverticulosis, Crohn’s disease, irritable bowel syndrome, cholelithiasis, cholecystitis, chronic gastritis, diverticulitis, undefined colitis, chronic hepatitis

Dietary interview disclosed that the adherence to gluten-free diet was not as good in the DM1 group as in the DM2 and CD groups (Table 3). This was also supported by the higher prevalence of EmA positivity in the DM1 group while on dietary treatment.

### Histological recovery on a gluten-free diet

A repeat biopsy on a gluten-free diet had been taken after a median of one year (range 0.5–41 years) in 51 % of the subjects in the DM1 group, 47 % in the DM2 group and 62 % in the CD group. Altogether 65 % of the patients in the DM1 group were found to have fully recovered small-intestinal mucosa, 12 % had partial villous atrophy and 12 % subtotal/total villous atrophy on diet; the corresponding figures in the DM2 group were 44, 50 and 6 % and in the CD group 71, 24 and 5 %, respectively (*p* = 0.059).

## Discussion

A novel finding of the present study is that DM1 is markedly overrepresented especially in men with celiac disease, whereas the prevalence of DM2 was practically similar to that of the Finnish population in general. Furthermore, concomitant DM1 or DM2 predispose celiac disease patients to severe comorbidities, and DM1 also to lower adherence to the gluten-free diet.

Here the overall prevalence of DM1 among celiac patients over 30 years of age was 3.8 %. This is significant overrepresentation compared to the population even in Finland where the incidence of DM1 is one of the highest in the world [12]. In contrast to our study, the great majority of the previous studies investigating the association between celiac disease and DM1 have been conducted in children [13, 14] of whom the characteristics of the concomitant diseases could be different. In addition, opposed to our design, the earlier studies have mainly investigated the prevalence of celiac disease in diabetic patients [15, 16]. Another interesting finding of the present study was the substantially higher prevalence of DM1 among men with celiac disease (8.0 %) compared with the corresponding women (1.8 %). Even though, unlike most other autoimmune diseases, DM1 is known to be in general more common in men [17, 18], this aspect has not been studied in celiac patients before, and the gender difference was surprisingly large. Interestingly, it has been observed that the male-to-female ratio of DM1 ratio differs between age groups [17], and also that it is higher in countries where DM1 is common [18]. The latter might partly explain the high number of DM1 men in our study population, but further studies are needed to fully explain these gender discrepancies. The prevalence of DM1 was also almost five times higher in celiac disease men aged 30–64 years compared with men being 65 years or more. The lower DM1 prevalence among older celiac patients could be due to increased mortality in DM1 [16]. Accordingly, it has been shown that co-existing celiac disease of more than 15 years increases the risk of death in DM1 patients compared to patients with only DM1 [19, 20]. The higher prevalence of DM1 in younger men here could also be partly due to increasing incidence of DM1 over time at population level [21].

The overall prevalence of DM2 in our celiac disease cohort was at the same level as in Finnish adults in general. Further, the only noticeable difference in the subgroup comparisons was the lower prevalence of DM2 in celiac men aged ≥65 years compared with the population. Our results differ from those observed by Kabbani and colleagues, who recently found the prevalence of DM2 to be more than three times lower in celiac disease patients than in the US population [6]. One explanation for the discrepant results could be differences in the disease presentation, as in Finland celiac patients have in general rather short diagnostic delay and mild clinical picture, whereas in US patients the classical malabsorptive disease with weight loss is still common [22, 23]. Finnish patients are usually normal weight or even overweight at diagnosis and it might be argued that they thus have “normal” risk for DM2 [24]. However, in the US study the protective effect of celiac disease was observed even after controlling for malabsorption, symptoms and BMI, indicating that there are other reasons for the different results. Another possibly affecting factor is the selection of control group and the overall prevalence of DM2 in the population. For example, the US study comprised two separate control groups, including age-, sex-, and ethnicity-matched controls and subjects of National Health and Nutrition Examination Survey [25]. The prevalences of DM2 in these groups were 9.6 % and 9.8 %, respectively, while in our control group it was only 4.4 % in men and 3.0 % in women. Obviously there can also be differences in genetics, diagnostic criteria of DM2 and lifestyle, but the true role of these factors remains to be solved in future studies.

The clinical presentation of celiac disease was quite similar in three groups, the only significant difference being the markedly higher rate of screen-detected patients in the DM1 group. This is not surprising, as in Finland the increased risk of celiac disease in DM1 patients is well-known among physicians, and thus they are screened at low threshold [26]. Subjects in DM1 group also had their celiac disease diagnosed at younger age, which might similarly be due to active screening. As expected, the presence of both concomitant DM1 and DM2 significantly increased the prevalence of coronary artery disease and hypertension in celiac disease patients compared with those being non-diabetic. This is in agreement with previous finding by Pitocco and co-workers that concomitant DM1 increases the risk of sub-clinical atherosclerosis in celiac disease [27]. However, since we did not have specific non-celiac DM1 or DM2 control groups, it remains unclear whether celiac disease truly has an additional effect to the risk of cardiovascular complications, or whether their increased prevalence are caused solely by the concomitant diabetes. The prevalence of thyroidal diseases was also higher in celiac patients with DM1 compared with other groups even after controlling for age and gender. This was also quite expected, as the association of autoimmune thyroidal diseases with both DM1 and celiac disease has been well-established [28, 29]. These disease associations are important to remember when treating patients with simultaneous celiac disease and diabetes.

Adherence to the gluten-free diet was significantly lower among celiac disease patients with a concomitant DM1 compared with the two other study groups. This result from interviews was further confirmed by the higher proportion of EmA-positivity in the DM1 group while on the diet. The lower adherence is in line with previous studies where the dietary adherence has also been relatively low among patients with the double-diagnosis of CD and DM1 [30, 31]. Celiac disease patients with a concomitant DM1 may find it more difficult to cope with the demanding diet while already needing to concentrate to continuous daily glucose level monitoring, carbohydrate calculation and insulin dosing. Moreover, as seen in here and previously [32], celiac patients with concomitant DM1 are often screen-detected and therefore have possibly only mild or no apparent symptoms, which might further decrease the motivation to adhere to expensive and socially restrictive gluten-free diet. There is no reason to believe that the maintenance of strict diet is less important in celiac patients with diabetes that in celiac disease altogether. These patients may need individualized support for the dietary treatment, and also careful follow-up to ensure coping with the increased disease burden. Accordingly, we have earlier shown that rigorous dietary counselling in patients with celiac disease and concomitant DM1 improves the dietary adherence to gluten-free diet [30]. Interestingly, despite the poorer adherence, DM1 patients did not show significantly inferior mucosal recovery after a median of one year on a gluten-free diet. Nevertheless, they had the highest percentage of subtotal or total villous atrophy while on diet. Furthermore, notwithstanding the apparently equal dietary adherence, there was also a trend for slower histological recovery among subjects in the DM2 group compared with those in the CD group. Further studies are needed to establish whether concomitant diabetes predisposes celiac disease patients to slower mucosal recovery or even persistent villous atrophy on a gluten-free diet.

Major strengths of the present study are the high number of biopsy-proven celiac disease patients with heterogeneous clinical presentation and the representative control population acquired from a nationwide public health study. In addition, we were able to gather abundant clinical and histological information from each patient. Major limitations were the retrospective study design and the lack of specific information on the disease profile of DM1 and DM2. Further, the non-celiac diabetic control groups would have been needed to estimate the role of concomitant celiac disease in the increased risk of cardiovascular complications. The fact that repeat biopsy on a gluten-free diet was taken less often from DM1 and DM2 patients may cause selection bias. It would have also been very interesting to see whether there are differences between the groups in overall mortality, but unfortunately we did not have this data systemically collected. In any case, the cross-sectional study design and a relatively short follow-up time in most of the patients might have made these results somewhat unreliable. Finally, there was no exact data when the diabetes diagnosis was made with respect of celiac disease diagnosis.

## Conclusions

To conclude, in Finnish adult population, DM1 was significantly overrepresented in celiac disease, whereas the prevalence of concomitant DM2 was at the same level as in the population. Clinicians should be aware that that the double diagnosis of celiac disease and either DM2 or DM1 predisposes the patients to severe co-morbidities. Celiac disease patients with concomitant DM1 have increased risk for dietary lapses and possibly also to poorer histological recovery, and require therefore specifically-tailored support and careful follow-up.

## Abbreviations

DM1, type 1 diabetes; DM2, type 2 diabetes; EmA, endomysial antibodies
